# Stem cell therapy for ischemic stroke: neuroimaging approaches and evidence from a systematic review

**DOI:** 10.3389/fneur.2026.1718086

**Published:** 2026-02-10

**Authors:** Bin Jiang, Moss Zhao, Elizabeth Tong, Yongkai Liu, Ates Fettahoglu, Wen-Kai Weng, Michael E. Moseley, Max Wintermark, Gary K. Steinberg, Greg Zaharchuk

**Affiliations:** 1 Department of Radiology, School of Medicine, Stanford University, Stanford, CA, United States; 2 Department of Neurosurgery, School of Medicine, Stanford University, Stanford, CA, United States; 3 Department of Medicine - Med/Blood and Marrow Transplantation, School of Medicine, Stanford University, Stanford, CA, United States; 4 Department of Neuroradiology, MD Anderson Center, The University of Texas, Houston, TX, United States

**Keywords:** brain, imaging, ischemic stroke, MRI, PET, stem cell

## Abstract

**Purpose:**

Cell-based therapy is a promising approach for ischemic stroke treatment. This systematic review and meta-analysis aimed to consolidate clinical evidence on the use of neuroimaging to evaluate stem cell therapy across all stages of stroke recovery.

**Methods:**

A systematic search was conducted in 5 databases in July 2025. They were included if neuroimaging analysis, regardless of cell source, route administration or dosage were reported. The level of evidence and risk of bias were assessed using the ROB-2 or ROBINS-I tool. Imaging data from all included articles were extracted, and randomized-effect meta-analyses were performed when two or more outcomes were available for any reported imaging parameter.

**Results:**

Thirty articles were included in the systematic review, of which four were eligible for meta-analysis. Meta-analysis of subacute stroke patients revealed no significant differences in infarct volume reduction at 3 months (SMD = −0.50; 95% CI: −1.15 to 0.51; *p* = 0.13; *I*^2^ = 63%) or 1 year (SMD = −1.02; 95% CI: −3.63 to 1.60; *p* = 0.45; *I*^2^ = 92%) between treatment and control group. Chronic stroke patients exhibited less overall volume loss. There was a trend toward improved white matter recovery and motor cortex activity, reflected in increased DTI and fMRI parameters. SPIO-labeled autologous stem cells recently proved safe in patients, with T2* imaging showing engraftment and migration.

**Conclusion:**

Advanced neuroimaging offers a valuable non-invasive tool for assessing the effects of stem cell therapy in ischemic stroke. However, substantial heterogeneity in imaging protocols and reporting limits cross-study comparisons. Standardization of neuroimaging methodology is essential to advance future research and clinical translation.

## Introduction

Stroke remains one of the leading causes of death and long-term disability worldwide. Among its subtypes, ischemic stroke accounts for the vast majority of cases, arising from obstruction of cerebral blood flow and subsequent brain tissue damage. The growing global burden is striking: from 1990 to 2019, the incidence of ischemic stroke increased by 70% and prevalence by over 100%, with parallel rises in mortality and disability (1). These statistics underscore the urgent need for more effective therapeutic strategies.

Currently, the most effective treatments for ischemic stroke are reperfusion-based interventions, including intravenous thrombolysis and endovascular therapy (EVT). While these approaches can restore blood flow and improve outcomes, their clinical application is limited by narrow therapeutic windows, strict eligibility criteria, and incomplete functional recovery in many patients (2). As a result, a substantial proportion of stroke survivors are left with persistent neurological deficits, highlighting the unmet need for restorative therapies that extend beyond the acute treatment window.

Stem cell therapy has emerged as a promising approach in this context. Building on the 2012 Nobel Prize–winning discovery that somatic cells can be reprogrammed into pluripotent stem cells, preclinical and clinical studies have explored the potential of stem cells to enhance brain repair after ischemic stroke. These studies suggest that stem cells may exert beneficial effects through multiple mechanisms, including promoting neurogenesis, angiogenesis, and synaptic plasticity, as well as modulating neuroinflammation and immune response (3–5). Clinical trials to date have demonstrated safety and feasibility, with early signals of efficacy across different stem cell types, transplantation routes, and stages after stroke onset (6–9).

Despite these advances, critical challenges remain in optimizing stem cell therapy for stroke, particularly in objectively assessing therapeutic effects in the human brain. Neuroimaging offers tools to address this need, providing noninvasive biomarkers of structural and functional brain changes. Techniques such as diffusion tensor imaging (DTI), functional MRI (fMRI), perfusion imaging, and positron emission tomography (PET) can reveal the mechanisms of action, monitor brain repair, and potentially predict clinical outcomes. While prior reviews have summarized stem cell mechanisms, delivery methods, and safety, comparatively little attention has been given to the role of neuroimaging in this field (2, 10–14).

The aim of this systematic review is therefore to synthesize current evidence on structural and functional neuroimaging of stem cell therapy in ischemic stroke. By focusing on imaging biomarkers, we highlight how neuroimaging contributes to understanding treatment mechanisms, evaluating efficacy, and guiding the future development of regenerative therapies for stroke patients.

## Methods

Ethics approval by an Institutional Review Board (IRB) was not required for this systematic review.

### Protocol and registration

The search strategy and written methodology were developed with the assistance of the medical librarians, following the Preferred Reporting Items for Systematic Reviews and Meta-Analyses (PRISMA) (15), the Peer-Review of Electronic Search Strategies (PRESS) (16), the National Academies (IOM) Standards for Systematic Reviews (17), and the Cochrane guidelines (18).

### Inclusion and exclusion criteria

Studies were included if they were published in a peer-reviewed journal, evaluated ischemic stroke in an adult population, administered a specific type of stem cells or stimulating factors for stroke, and included results based on one or more evaluations of functional imaging parameters, including MRI or PET (Table 1). Exclusion criteria were studies that were not full research articles, animal studies, dissertations, or review articles. Additionally, studies focusing on populations with hemorrhagic stroke or strokes from sickle cell or moyamoya diseases were excluded. This selection was chosen to enhance the focus on imaging related to arterial ischemic infarcts.

**Table 1:** Inclusion and exclusion criteria for studies identified in the search (PICOS).

Inclusion criteria	Exclusion criteria
Population: Adult (age 18+) Diagnosis: confirmed ischemic stroke (irrespective of stage)	Population: Pediatric patients Animal studies Diagnosis of: Hemorrhagic stroke Stroke from sickle cell disease/Moyamoya disease
Intervention: Treated with stem cells/stimulating factors AD-MSCs: amniotic-derived mesenchymal stem cells BMMSCs: bone marrow mesenchymal stem cells HNSC: human neural stem cell MSCs: mesenchymal stem cells NPCs: neural progenitor cells NSPCs: neural stem/progenitor cells OECs: olfactory ensheathing cells PBSCs: peripheral blood stem cells SCs: Schwann cells UCB: umbilical cord blood UCMSCs: umbilical cord mesenchymal stromal cells Dental pulp stem cells CD34 + cells G-CSF: Granulocyte colony-stimulating factor	Intervention: Not treated with mentioned stems/stimulating factors Publications without enough imaging and outcome data or not peer-reviewed: Abstracts Dissertations Book chapters Systematic reviews Commentary Interviews Study protocols PhD theses
Comparisons: With control group and/or With baseline and/or With contralesional hemisphere and/or Correlation with clinical biomarkers	No quantitative comparison /longitudinal assessment /clinical correlation.
Outcome: Imaging-based quantitative measures, including: Structural MRI (e.g., infarct volume, T2Flair high signal) DTI (e.g., FA, MD) Perfusion imaging (e.g., CBF, CBV, MTT) fMRI (task-based or resting-state) MR spectroscopy (metabolite ratios) SPIO labeled imaging PET (e.g., SUVr)	No imaging or lower-resolution / non-quantitative imaging modalities (e.g., CT, SPECT, ultrasound)

### Information sources and search strategy

MEDLINE, EMBASE, Scopus, Cochrane Central Register of Controlled Trials and Web of Science were searched by a medical librarian (EW). To identify any missing reports, we also hand-searched the references lists from included studies and identified articles meeting the inclusion criteria, contacting authors and experts, and examining related articles in PubMed and Google Scholar. An updated search was conducted in July 2025. Complete, reproducible search strategies for each database are provided in the Supplementary materials.

### Study selection and data extraction

All titles, abstracts, and full manuscripts underwent review by two unique individuals within the authorship team, utilizing Covidence systematic review software. In cases of discrepancies, two authors (BJ, MZ) conducted a joint review to reach a final decision regarding study inclusion. To enhance transparency, the predefined PICOS-based inclusion and exclusion criteria (Table 1) were applied consistently throughout title/abstract and full-text review. After full-text screening, we collected the data using a data extraction form. Data collection included: stage of ischemic stroke (acute [<7 days], subacute [1 ~ 12 weeks] or chronic [>3 months]), route, dosage, and type of stem cells introduced, baseline NIHSS, the number of subjects in the intervention and the control group (if present), infarct size and how it was reported (volume, percentage, and etc.), DTI parameters (FA, MD, and etc.), functional MRI, PET changes, and other imaging parameters (such as SPIO labeled T2*).

### Risk of bias assessment and level of evidence

Two authors (BJ, YL) evaluated the risk of bias using the ROB-2 (Cochrane risk-of-bias tool) for the randomized control studies (19), and ROBINS-I (Risk of Bias in Non-Randomized Studies of Interventions) tool for non- randomized control studies (20). The following domains were assessed: confounding, selection of participants, classification of interventions, deviations from intended interventions, missing data, measurement of outcomes, selection of reported results, and overall risk of bias. The risk of bias was then classified as high, moderate, or low according to the ROB-2 and ROBINS-I tool. The corresponding author was contacted to retrieve missing data. To grade the overall strength of evidence from the included studies, the level of evidence of each included study was assessed by The Oxford Centre for Evidence-Based Medicine Levels of Evidence and scored accordingly (CEBM) (21).

### Reporting bias assessment

If at least 10 studies were available for an outcome, we planned to assess reporting bias using funnel plots.

### Statistical analysis

We employed a random-effects meta-analysis to combine the study results. Meta-analyses were conducted using Review Manager version 5.4 software (RevMan 2020). For continuous variables, we computed the standard mean difference (SMD) and 95% confidence interval (CI). In cases where numerical outcome data were not directly reported in the original publications, graphical data were extracted. We obtained these values either by contacting the corresponding authors or, when necessary, by digitizing published figures using Web Plot Digitizer^1^ (22). This ensured inclusion of studies that otherwise lacked extractable numerical data. Heterogeneity was quantified with the *I*^2^ statistic, with values of 25, 50, and 75% considered low, moderate, and high, respectively. A two-tailed *p* value < 0.05 was considered statistically significant.

## Results

### Search results

**Figure 1:**
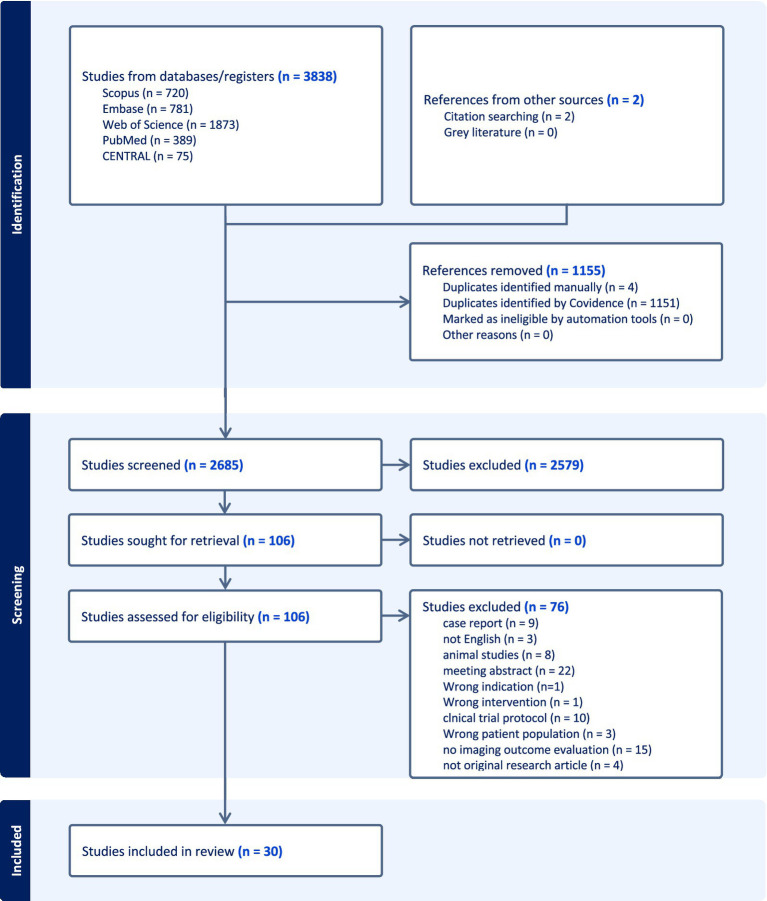
PRISMA flowchart illustrating the process of selecting studies included.

A total of 3,838 articles from 5 databases were initially identified. After removing duplicates, 2,685 articles remained. Full-text review was completed on 106 studies, and 30 studies were included in this review (Figure 1). Studies were excluded for the following reasons: (1) not peer-reviewed (*n* = 45); (2) no imaging to outcome analysis or insufficient imaging data (*n* = 15); (3) ineligible population (*n* = 11); (4) non-English (*n* = 3); (5) wrong intervention (*n* = 1); (6) wrong indication (*n* = 1).

### Characteristics and quality of evidence of included studies

Of the 30 included studies, 22 were clinical trials, with 8 studies involving sub-analyses of the same study. These studies were conducted in various regions: 4 in North America, 5 in Europe, 12 in Asia, and 1 in Africa (Table 2). All studies incorporated MRI or PET. Among them, 16 studies compared infarct sizes between baseline and the latest available follow-up based on T1-weighted images, 6 included the identification of abnormal high signals on T2 FLAIR images immediately after treatment, 9 included analysis of DTI images, 4 involved functional MRI analysis, 2 studies analyzed perfusion MRI changes, and 5 included PET analysis.

**Table 2:** Basic characteristics of included studies.

Study	Clinical trial	Stage of stroke	Intervention (# subjects)	Control (# subjects)	Route of introduction	Cell type	Infarct size	T2 Flair	DTI	fMRI	Perf-usion	PET	Others	level of evidence†
Kawabor et al. (38)	N-RCT	Subacute (9 ~ 12 day)	7	None	IC	Autologous bone marrow derived stromal cells		+					SPIO-T2* /SWI	4
Moniche et al. (30)	RCT (IBIS)	Acute (<7 Days)	38	36	IA	Autologous bone-marrow derived mononuclear stem cell	+							1B
Chiu et al. (34)	N-RCT	Chronic (1.6, 6, 2.4 years)	3	None	IC	Adipose tissue derived stem cells	+	+						4
Bang et al. (40)	RCT (STARTING-2)	subacute (<=90 days)	39	15	IV	Autologous bone-marrow mesenchymal stromal cell			+					1B
Lee et al. (44)	RCT (STARTING-2)	Subacute (<=90 days)	31	13	IV	autologous bone-marrow mesenchymal stromal cell			+	+				1B
Law et al. (21)	RCT	Subacute (<2 months)	9	8	IV	Autologous bone-marrow derived mesenchymal stromal cells	+							1B
Haque et al. (27)	N-RCT	Acute (≤72 h)	25	20	IV	Autologous bone-marrow derived mononuclear stem cell	+		+					2B
Jaillard et al. (45)	RCT (ISIS-HERMES)	Subacute (31 ± 7 days)	16	15	IV	Autologous bone-marrow derived mesenchymal stromal cell				+				2B
Haque et al. (28)	N-RCT	Acute (72 h)	9	None	IV	Autologous bone-marrow derived mononuclear stem cell	+						MRS	2B
Zhang et al. (33)	N-RCT	Chronic (3–24 months)	9	None	IC	NSI-566 cells		+	+		+	+		4
Vahidy et al. (39)	N-RCT (SIVMAS)	Acute (≤72 h)	25	None	IV	Bone-marrow derived mononuclear stem cell			+					2B
Steinberg et al. (37)	N-RCT	Chronic (6–60 months)	18	None	IC	SB623 cells		+						4
Kalladka et al. (46)	N-RCT (PISCES)	chronic (6–60 months)	7	None	IC	CTX0E03 cells				+				4
Ghali et al. (24)	N-RCT	Subacute (1-12 week)	21	18	IA	Autologous bone-marrow derived mononuclear stem cell	+							2B
Kalladka et al. (35)	N-RCT (PISCES)	Chronic (6–60 months)	11	None	IC	CTX0E03 cells		+	+					4
Steinberg et al. (36)	N-RCT	Chronic (6–60 months)	18	None	IC	SB623 cells		+						4
Wanamake et al. (32)	N-RCT	Chronic (6–60 months)	5	5	IC	Mesenchymal stem cells	+							2B
Taguchi et al. (49)	N-RCT	Acute (≤10 days)	7/12	0/59	IV	Autologous bone-marrow derived mesenchymal stromal cell						+		4
Banerjee et al. (29)	N-RCT	Acute (<7 days)	5	None	IA	Autologous CD34 + selected stem/progenitor cell	+							4
Prasad et al. (25)	RCT	Subacute (7–30 days)	57	60	IV	autologous bone-marrow derived mononulcear cells	+							1B
Chen et al. (41)	RCT	chronic (6-60 months)	15	15	IC	GCS-F + autologous peripheral blood hematopoietic stem cells			+					2B
England et al. (23)	RCT	Subacute (3-30 days)	14/40	6/20	subcutaneous	G-CSF	+							1B
Bhasin et al. (43)	N-RCT	Chronic (3-24 months)	12	12	IV	Autologous bone-marrow derived mesenchymal stromal cell			+	+				2B
Boy et al. (62)	N-RCT	Acute (<12 h)	18	None	subcutaneous	G-CSF	+							4
Honmou et al. (42)	N-RCT	subacute and chronic (36-133 days)	12	None	IV	Autologous bone-marrow derived mesenchymal sromal cell	+		+		+			4
Savitz et al. (63)	N-RCT	Acute (1 ~ 3 day)	10	None	IV	Autologous bone-marrow derived mesenchymal stromal cell	+(IER)							4
Shyu et al. (48)	RCT	Acute (<7 days)	7	3	subcutaneous	G-CSF	+					+		2B
Bang et al. (31)	RCT	chronic (41-61 days)	5	5/25	IV	Autologous bone-marrow derived mesenchymal stromal cell	+							2B
Meltzer et al. (47)	N-RCT	Chronic (6–72 months)	11	None	IC	LBS-Neurons						+		4
Kondziolka et al. (50)	N-RCT	Chronic (6–72 months)	11	None	IC	LBS-Neurons	+					+		4

NSI-566 cells: human neural stem cells clonally derived from human fetal spinal cord without genetic modification; SB623 cells: allogeneic modified bone marrow–derived mesenchymal stem cells with a plasmid vector encoding the human Notch-1 intracellular domain; CTX0E03 cells: human neural stem cells clonally derived from human fetal cortical epithelium; LBS-Neuron: produced from the NT2/D1 human precursor cell line. IC: Intracerebral. RCT: randomized, controlled clinical trial. N-RCT: not randomized, controlled clinical trial. †The levels of evidence, as defined by the Oxford Centre for Evidence-Based Medicine (21), range from Level 1A, which includes systematic reviews of well-conducted randomized controlled trials (RCTs), to Level 5, which consists of expert opinions without critical appraisal. Intermediate levels include individual RCTs (Level 1B), systematic reviews of cohort studies (Level 2A), individual cohort studies or low-quality RCTs (Level 2B), systematic reviews of case–control studies (Level 3A), individual case–control studies (Level 3B), and case series or poor-quality observational studies (Level 4). Each level reflects the methodological rigor and reliability of the evidence provided.

There were 10 randomized controlled clinical trials, 4 non-randomized controlled clinical trials, 14 case series, and 2 observational case–control study that were included in this systematic review. These reflected a mixture of Class 1B (*n* = 6), Class 2B (n = 10), and Class 4 (*n* = 14) studies. Specifically, three studies reported on infarct size 90 days after treatment (23–25), and two studies provided data at the 1-year mark (24, 26).

### Risk of bias within and across studies

**Figure 2:**
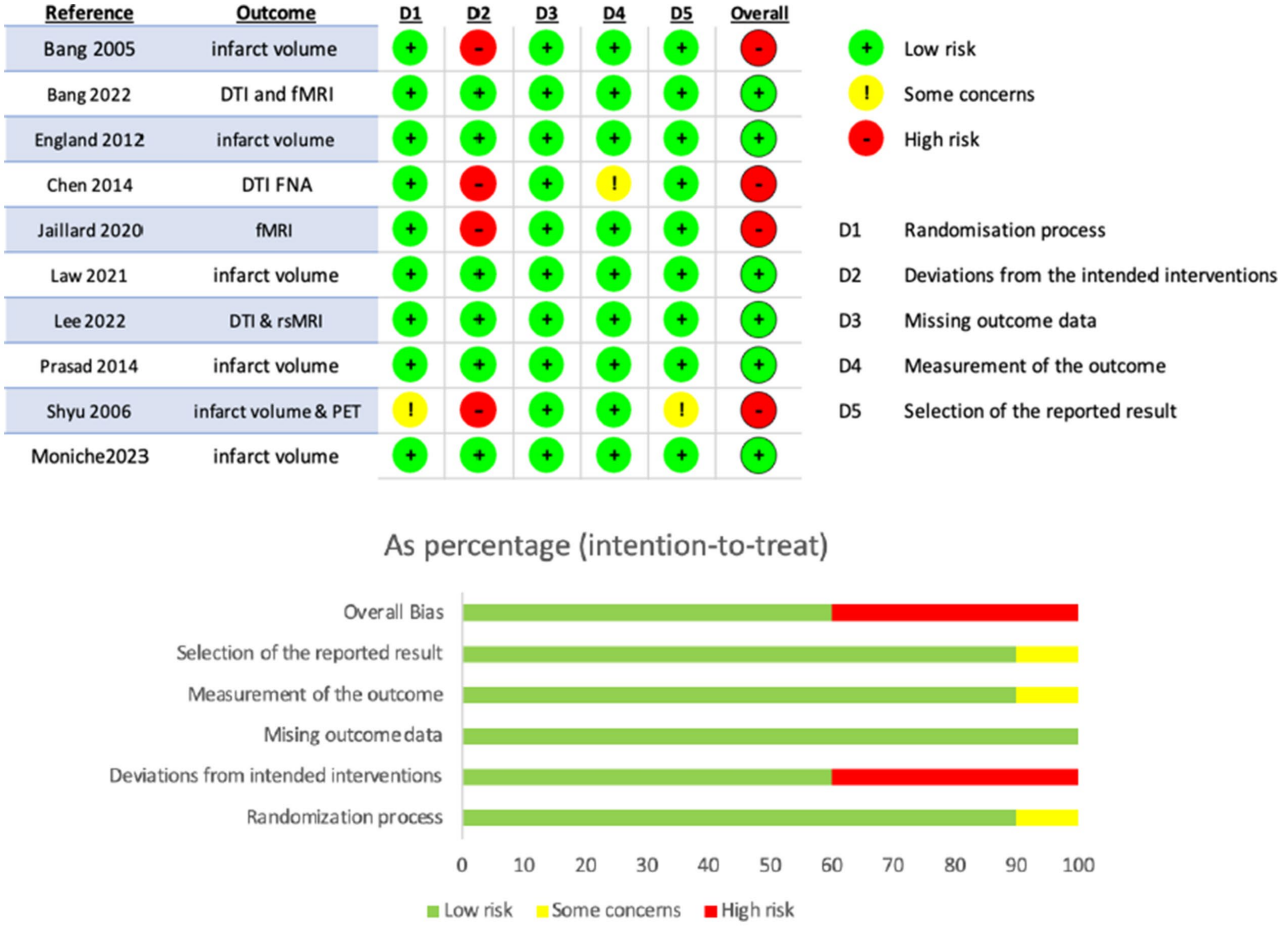
Summary of risk of bias assessment as determined with the RoB-2 tool in the 10 randomized controlled trials.

**Figure 3:**
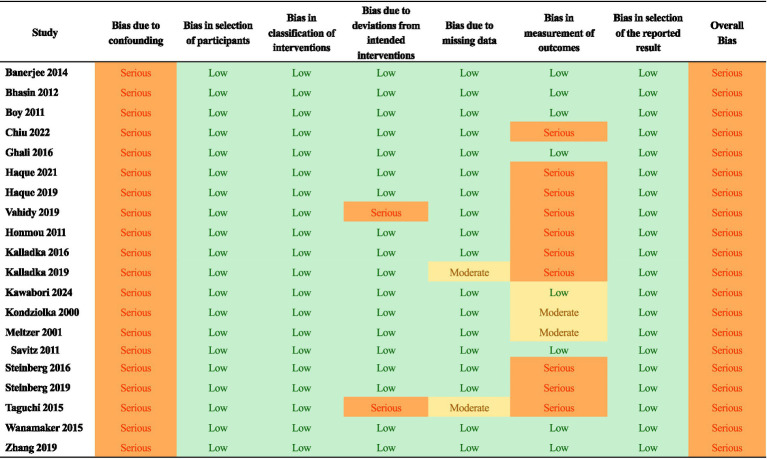
Summary of risk of bias assessment in the non-randomized clinical trials as determined with the ROBINS-I tool.

The included studies showed varying levels of bias (Figures 2, 3). Among the randomized controlled trials, four were rated as high risk of bias and six as low risk. All non-randomized studies were judged to have serious risk of bias, primarily due to confounding, such as differences in rehabilitation received by participants. The absence of control groups was especially common in intracerebral implantation trials, where sham procedures were not feasible. No study was rated as having critical or unclear risk of bias. Given this review’s focus on imaging, the lack of blinding was identified as the most frequent limitation, particularly in studies where regions of interest were manually delineated.

### Summary of imaging findings

- Infarct volume changes

**Figure 4:**
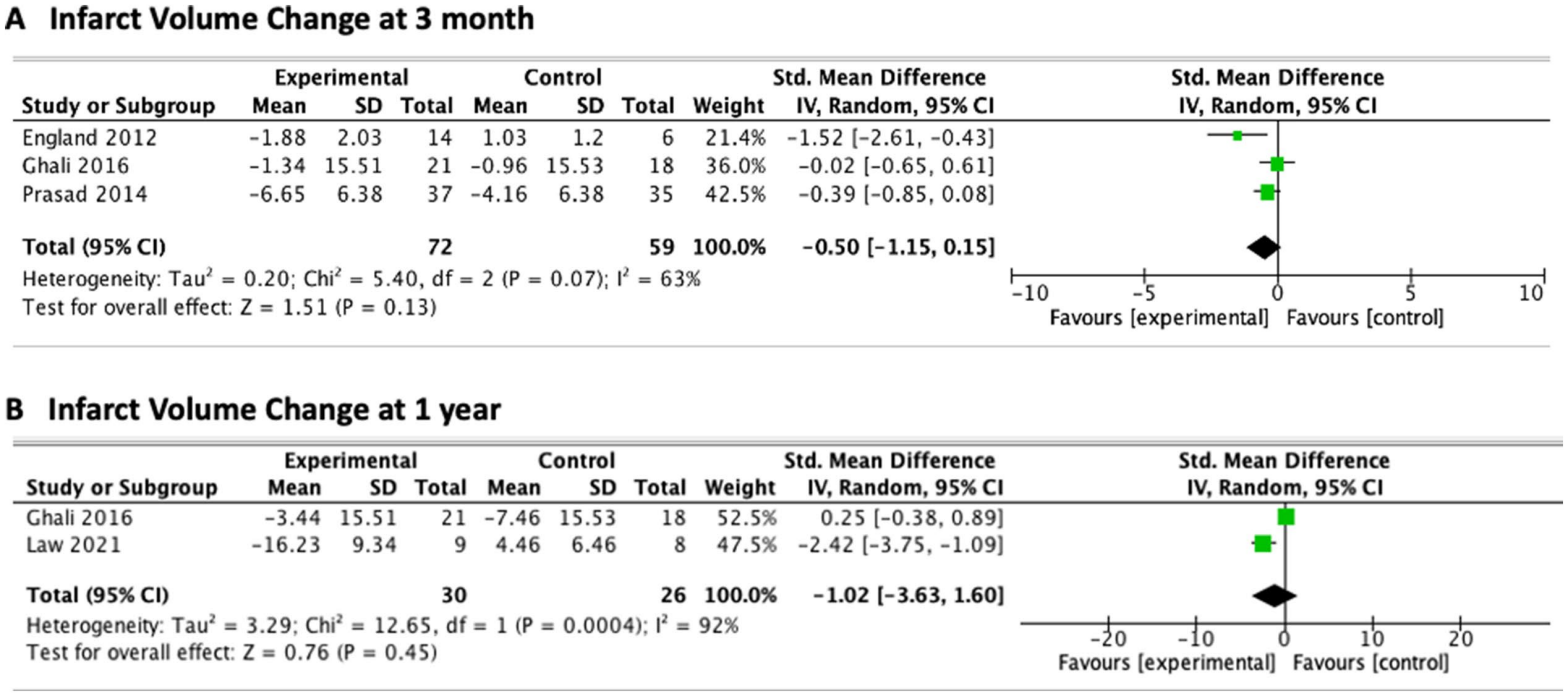
Meta-analysis of infarct volume changes among subacute ischemic stroke patients undergoing therapy. **(A)** Infarct volume change at 3 months after treatment. **(B)** Infarct volume change at 1 year after treatment. Forest plots display standardized mean differences (SMDs) with 95% confidence intervals using a random-effects model, comparing intervention and control groups.

Although 16 studies investigated infarction volume (Table 2) on structural T1 images, quantitative data were available from 9 studies (23–31). Volume change analysis was available in 4 studies, all conducted in subacute stroke patients (23–26). The treatment group (72 subjects) showed non-significant volume reduction compared to control (59 subjects) group (SMD –0.50; 95% CI [−1.15, 0.51]; *p* = 0.13; *I*^2^ = 63%; Figure 4A). No significant difference was found between the two groups at 12 months which was derived from 2 studies (24, 26) (SMD –1.02; 95% CI [−3.63, 1.60]; *p* = 0.45; *I*^2^ = 92%; Figure 4B). For chronic stroke, 2 studies showed a trend toward less overall volume loss after mesenchymal stem cell implantation by either measuring the secondary dilation of the adjacent ventricle or using automated brain volume quantification (31, 32).

- T2 FLAIR

Six studies (*n* = 41) using intracranial administration in chronic stroke patients reported hyperintense signals on T2 FLAIR (33–37). Of these, 21/41 patients exhibited hyperintensity, most prominently in the premotor cortex.

In general, signals appeared as early as 3 days post-implantation, peaked at 7–14 days, and resolved within 1–2 months or, in some cases, persisted up to 24 months (35, 36, 38). One study quantified lesion size and found significant correlations with clinical improvements in ESS, NIHSS, F-M total score, and FMMS at 1 year, and in ESS and NIHSS at 2 years (36, 37). The transient FLAIR hyperintensity, showing negative diffusion appearance, was interpreted as vasogenic edema, possibly reflecting blood–brain barrier disruption due to neurotrophic factor release (e.g., VEGF) or inflammatory response.

- DTI

Nine studies investigated DTI, seven of which focused on the corticospinal tract (CST) (27, 33, 35, 39–44). Five studies reported positive quantitative findings (27, 39–41, 44). General patterns included: an initial FA decrease followed by gradual recovery at 1–2 years in acute stroke patients treated with stem cells (27, 39), and FA preservation in subacute patients compared with controls (44).

FA values showed subtle changes over time, detectable at the hundredth decimal place, prompting the use of relative FA (rFA = ipsilesional/contralesional) as a more robust metric (27). Haque et al. found that rFA of the rostral pontine CST negatively correlated with NIHSS score at 1, 3, and 12 months (27). Chen introduced FNA (fiber numbers asymmetry) from tractography, noting increased fiber numbers correlated with lower NIHSS scores in treated patients (41). ROIs around the needle track found a non-significant FA increase near the putamen and cortex (35).

- Functional MRI

Four studies were identified (43–46), comprising two motor task and two resting-state fMRI studies.

Motor task fMRI: Both studies (intravenous route, chronic or subacute stroke, with control groups) demonstrated significant improvement in primary motor cortex (BA4) activation (43, 45). Supplementary motor cortex (BA6) also showed improvement at 2 months but not at 6 months when measured by laterality index (43).

Resting-state fMRI: One study (*n* = 31 treated, *n* = 13 controls) showed increased ipsilesional connectivity at 90 days post-treatment, with no changes in interhemispheric connectivity and only a trend toward increased network efficiency and density (44). Another small study (*n* = 7, no controls, intracerebral putamen injection) reported increased connectivity with the bilateral caudate and contralateral thalamus, and decreased connectivity with the ipsilateral parietal lobe at 1 month. Connectivity changes correlated with spasticity scores, and overall rs-fMRI connectivity increased across six deep gray matter regions at 1 month (46).

- PET

Five studies were included, all using automated image registration and processing (33, 47–50). FDG-PET (3 studies) showed increased lesion-to-perilesional SUVr at 10–20 months and beyond (33), with positive correlations between peri-lesional metabolism and clinical outcomes (15, 47, 48). [^15^O]H_2_O PET demonstrated increased contralesional rCBF and bilateral regional metabolic rate of oxygen consumption (rCMRO2) at 6 months (49).

Crossed cerebellar diaschisis was consistently observed pre-transplantation. One study quantified a reduction post-transplantation but without statistical significance (33), while another described qualitative reduction only (49). Meltzer et al. reported correlations between metabolic recovery and NIHSS/ESS improvements (47).

- Perfusion

MRI perfusion analysis was identified in 2 studies (33, 42). ASL perfusion study demonstrated significant lesion-to-perilesional CBF ratio differences at 10–20 months and > 20 months, but not at 6–10 months (33). One single-subject was found to have a CBF increase in peri-infarct tissue 7 days post-injection (42).

- Other Modalities

SPIO Tracking: SPIO (superparamagnetic iron oxide) labeling has long been used in preclinical animal models to monitor the migration and survival of transplanted stem cells. Recent clinical evidence has now demonstrated its feasibility in humans (38). Transplanted cells were initially detected at the injection site, with signal intensity declining within the first month. New hypointense T2* signals emerged in the peri-infarct region between 1 and 6 months and persisted for at least 1 year. These *de novo* signals, predominantly located near the subventricular zone, were interpreted as migrated cells.

Magnetic Resonance Spectroscopy (MRS): One study (*n* = 8) found significant increases in N-acetylaspartate (NAA) between 3 and 6 months, with NAA positively correlating with NIHSS at 3 months. This temporal metabolite changes were suggested to reflect therapeutic effects (28).

## Discussion

This systematic review synthesizes current evidence on the application of neuroimaging in evaluating stem cell therapy for ischemic stroke. While clinical outcomes remain the primary endpoints in stroke trials, neuroimaging provides a complementary lens by uncovering biological processes that underlie recovery. These imaging-based findings are particularly valuable in early-phase studies, where treatment effects may be subtle or delayed, and can help identify biomarkers of therapeutic efficacy. Together, the reviewed evidence suggests that neuroimaging has the potential not only to measure outcomes but also to guide mechanistic understanding and optimize treatment strategies.

### Interpretation of imaging findings

Given that most spontaneous recovery occurs within the first 3–6 months post-stroke (51), our meta-analysis focused on subacute patients to minimize confounding from natural recovery (52). Despite this refinement, the pooled results demonstrated no statistically significant differences in infarct volume between treatment and control groups at both 3 and 12 months. This reinforces the recognition that infarct volume is an insensitive marker of stem cell effects. Our findings, therefore, emphasize the limited value of infarct volume as a surrogate endpoint for regenerative therapies and highlight the need to rely on advanced neuroimaging biomarkers that are more closely tied to neural repair and functional recovery.

Advanced modalities such as DTI, fMRI, and PET offer a more nuanced picture. DTI findings suggest that stem cell therapy may help preserve or restore microstructural integrity within the corticospinal tract, although absolute FA changes were minimal (53–55). The use of rFA and tractography-derived measures such as fiber number asymmetry reflects the field’s effort to detect subtle changes that may relate to motor recovery. However, heterogeneity in acquisition and processing methods remains a major barrier to interpretation, limiting the comparability of results across studies.

Functional and metabolic imaging extend these observations beyond the lesion site. Functional MRI studies demonstrated increased motor cortex activation and changes in interhemispheric connectivity, while PET revealed contra-lesional increases in perfusion and metabolism (49). Together, these findings support the concept of bilateral reorganization and neuroplasticity, consistent with preclinical evidence that stem cell therapy may facilitate network-level compensation rather than purely local repair (56–59). Such mechanisms may be critical for explaining functional recovery when direct cell replacement is unlikely.

The recent clinical study showed SPIO-labeled stem cell tracking is safe and feasible (38). Their imaging observations offer valuable insight into the spatiotemporal dynamics of cell therapy and may help clarify the underlying therapeutic mechanisms. The detection of *de novo* signal changes in peri-infarct regions up to 1 year highlights the potential for long-term cell survival and migration. Although preliminary, these findings underscore the value of imaging as a tool for monitoring cell fate and optimizing delivery strategies.

Findings across PET, ASL perfusion, and SPIO tracking converge on the peri-infarct region as an interesting target of stem cell–mediated repair. Rather than altering the infarct core, stem cell therapy appears to enhance perfusion and metabolism in the peri-lesional rim (PIR) while transplanted cells migrate and persist there for months, often near the subventricular zone. This pattern supports the concept of the PIR as a “receptive niche,” where viable tissue and microvasculature provide a substrate for angiogenesis, neuroplasticity, and synaptic remodeling. Such processes help explain why infarct volume remains insensitive to treatment effects. These insights suggest that future trials should prioritize PIR-specific imaging biomarkers, which may better capture the mechanisms of repair and their relationship to functional recovery.

Our synthesis based on current evidence shows that the value of neuroimaging depends not only on modality but also on timing. FLAIR and DTI can provide important information across phases, whereas functional and metabolic changes are best captured after 1 month when physiologic remodeling is most pronounced. SPIO tracking offers additional insights into cell fate during the first year. While dense longitudinal imaging would maximize detection of these processes, it is often impractical and costly. A more scientific and cost-effective approach may be to prioritize a few critical timepoints — for example, early subacute (safety and acute response), 3–6 months (network reorganization and perfusion changes), and 12 months (durability of effects). These strategically selected windows balance feasibility and may help optimize the design of future trials.

### Clinical implications for radiologists

As stem cell therapies move closer to clinical adoption, radiologists will increasingly encounter patients who have undergone these interventions. Characteristic findings may include early post-operative high T2 FLAIR signal and contrast enhancement along the needle track with gradual resolution (36), growth of new tissue inside the cavity (33); and less extensive volume loss on future follow-up studies (32). Complications such as subdural and epidural hematoma and parenchymal hemorrhage have been reported by other review articles (60, 61). If stem cells were labeled with supermagnetic iron nanoparticles, they may show dark signals on susceptibility-weighted or T2*-weighted images 24 h after introduction, which fade gradually after 1 month and migrate afterwards (38). Moreover, systemic distribution, such as spleen involvement after intravenous infusion, reinforces the need for whole-body perspectives when interpreting imaging in this setting (3). Awareness of these patterns will be helpful for distinguishing expected treatment effects from other pathologies.

### Need for imaging standardization and centralized processing

A major barrier to progress remains the lack of standardized imaging pipelines and reporting practices. To enable reproducibility and facilitate data pooling, future studies may consider adopt centralized processing frameworks such as fMRIPrep for functional MRI and QSIPrep for diffusion imaging and use shared anatomical templates and standardized data formats (e.g., BIDS). Incorporating automated analysis can reduce inter-rater variability and increase transparency, particularly in multicenter studies. Sharing raw data between groups may also increase the confidence in any reported results.

Several limitations must be taken into account. A notable challenge in this field is the heterogeneous reporting of neuroimaging characteristics post-treatment. The small sample size in the meta-analysis raises concerns about its validity and requires further validation. Many early-phase trials are small, so small-study bias is possible. These studies were included because they provided the only imaging data available, but their limited size reduces precision and generalizability and underscores the need for larger trials. Sub-analyses based on the route of introduction and correlations with clinical biomarkers were not feasible. The included studies exhibited bias, with most non-RCTs lacking a sham control group, highlighting the necessity for improved study designs to minimize biases and capture specific outcomes. Despite these limitations, this represents the most current systematic review and meta-analysis exploring the role of neuroimaging after stem cell treatment.

## Conclusion

This systematic review highlights the value of neuroimaging in advancing the evaluation of stem cell therapy for ischemic stroke. Although many uncertainties remain regarding the most sensitive imaging biomarker, current evidence suggests that infarct volume is an insensitive marker of stem cell effects, advanced imaging can detect subtle but biologically meaningful changes, particularly in the peri-infarct region and across brain networks. These findings are clinically relevant for radiologists, who may increasingly encounter post-therapy imaging features, and scientifically important for informing the design of future trials that incorporate imaging biomarkers as endpoints. To move the field forward, larger, well-controlled studies with standardized pipelines, blinded image analysis, and data sharing are essential. Collectively, our findings indicate that while much is still unknown, neuroimaging provides a critical window into how stem cell therapies may reshape the injured brain and guide the development of regenerative strategies.

## Data Availability

The original contributions presented in the study are included in the article/Supplementary material, further inquiries can be directed to the corresponding author/s.

## Footnotes

1
https://apps.automeris.io/wpd/

## Glossary

ROB-2: Risk of Bias
ROBINS-I: Risk of Bias In Non-Randomized Studies - of Interventions (ROBINS-I)
EVT: Endovascular therapy
DALY: Disability Adjusted Life Years
DTI: Diffusion tensor imaging
fMRI: Functional magnetic resonance imaging
PET: Positron emission tomography
PRISMA: Preferred Reporting Items for Systematic Reviews and Meta-Analyses
PICOS: Population/Patient, Intervention, Comparison, Outcome, and Study Design
SPECT: Single-photon emission computed tomography
CEBM: Centre for Evidence-Based Medicine
FA: fractional anisotropy
AD: Axial diffusivity
RCT: Randomized clinical trial
ESS: European Stroke Scale
NIHSS: National Institutes of Health Stroke Scale
F-M: Fugl-Meyer
FMMS: Fugl-Meyer motor scale score
CST: cortical spinal tract
PLIC: posterior limb of internal capsule
FNA: fiber numbers asymmetry
BA4: primary motor area
MI-4a: anterior BA4
MI-4p: posterior BA4
BA6: Supplementary motor cortex
FDG: 18F-fluorodeoxyglucose
SUV: Standardized uptake value
CBF: cerebral blood flow
rCMRO2: regional metabolic rate of oxygen consumption
OEF: oxygen extraction fraction
ASL: arterial spin labeling
