# Supplementary material for: Stem cell therapy for ischemic stroke: neuroimaging approaches and evidence from a systematic review
Source: Front Neurol. 2026 Feb 10;17:1718086. doi: 10.3389/fneur.2026.1718086 (PMC12931286; doi:10.3389/fneur.2026.1718086)
Supplement: Supplementary file 1 [file Table_1.docx]

Supplementary table1: Complete, reproducible search strategies for each database.

PUBMED: (("Stem Cells"[Majr] OR "stem cell"[tiab] OR "stem cells"[tiab]) OR (“cell transplantation”[mh] OR “stem cells”[mh] OR “cells/tr”[mh] OR “Stem cell”[tiab:~5] OR “stem cells”[tiab:~5] OR “progenitor cell”[tiab:~5] OR “progenitor cells”[tiab:~5] OR “embryo cell”[tiab:~5] OR “embryos cell”[tiab:~5] OR “embryonic cell”[tiab:~5] OR “embryonal cell”[tiab:~5] OR “embryo cells”[tiab:~5] OR “embryos cells”[tiab:~5] OR “embryonic cells”[tiab:~5] OR “embryonal cells”[tiab:~5] OR “fetal cell”[tiab:~5] OR “foetal cell”[tiab:~5] OR “Fetal cells ”[tiab:~5] OR “foetal cells”[tiab:~5] OR “umbilical cell”[tiab:~5] OR “bone marrow cells”[tiab:~5] OR “cord blood cell”[tiab:~5] OR “cord blood cells”[tiab:~5] OR “cell transplantation”[journal]) OR ((“neural stem” OR “mesenchymal stem” OR “mesenchymal stromal” OR “mononuclear” OR “mononuclear precursor” OR “Schwann” OR “olfactory ensheathing” OR “olfactory glia” or “oligodendrocyte precursor”) AND (cell OR cells))

AND

("Neuroimaging"[Majr] OR "Magnetic Resonance Imaging"[Mesh] OR "Tomography, X-Ray Computed"[Mesh] OR "Ultrasonography"[Mesh] OR neuroimaging OR MRI OR "magnetic resonance imaging" OR "CT scan" OR "CAT scan" OR "computerized tomographic imaging" OR "Xray tomography" OR ultrasonography OR ultrasound OR "Tomography, Emission-Computed, Single-Photon"[Mesh] OR "Positron Emission Tomography Computed Tomography"[Mesh] OR “Diffusion tensor imaging” OR "Diffusion Magnetic Resonance Imaging"[MeSH]OR “DTI” [MeSH] OR “functional MRI” ] OR “fMRI” [MeSH] OR “arterial spin labeling” OR “single delay ASL” OR “multi delay ASL” OR “perfusion imaging” OR “positron emission tomography” OR “single photon emission computed tomography” OR “PET scan” OR SPECT[ti])

AND

("Stroke"[Majr] OR “strokes” OR "Cerebrovascular Accident" OR "Cerebrovascular Accidents")

WOS: TI=("Stroke" OR “strokes” OR "Cerebrovascular Accident" OR "Cerebrovascular Accidents"

OR "Cerebrovascular Accidents") OR

TS=("Stroke" OR “strokes” OR "Cerebrovascular Accident" OR "Cerebrovascular Accidents")

AND

TI=(("Stem Cells" OR "stem cell" OR “cell transplantation” OR “stem cells” OR “Stem cell” OR “stem cells” OR “progenitor cell” OR “progenitor cells” OR “embryo cell” OR “embryos cell” OR “embryonic cell” OR “embryonal cell” OR “embryo cells” OR “embryos cells” OR “embryonic cells” OR “embryonal cells” OR “fetal cell” OR “foetal cell” OR “Fetal cells ” OR “foetal cells” OR “umbilical cell” OR “bone marrow cells” OR “cord blood cell” OR “cord blood cells”) **OR** (“neural stem” OR “mesenchymal stem” OR “mesenchymal stromal” OR “mononuclear” OR “mononuclear precursor” OR “Schwann” OR “olfactory ensheathing” OR “olfactory glia” or “oligodendrocyte precursor”) AND (cell OR cells)) OR SO=(CELL TRANSPLANTATION)

AND

TS=("Neuroimaging" OR "Magnetic Resonance Imaging" OR "Tomography, X-Ray Computed" OR "Ultrasonography" OR neuroimaging OR MRI OR "magnetic resonance imaging" OR "CT scan" OR "CAT scan" OR "computerized tomographic imaging" OR "Xray tomography" OR ultrasonography OR ultrasound OR "Single Photon Emission Computed Tomography Computed Tomography" OR "Tomography, Emission-Computed, Single-Photon" OR "Positron Emission Tomography Computed Tomography" OR “Diffusion tensor imaging” OR "Diffusion Magnetic Resonance Imaging" OR “DTI” OR “functional MRI” OR “arterial spin labeling” OR “single delay ASL” OR “multi delay ASL” OR “perfusion imaging” OR “positron emission tomography” OR “single photon emission computed tomography” OR “PET scan” OR SPECT)

EMBASE: ('cell transplantation'/exp OR 'cell transplantation' OR 'stem cell'/mj/exp OR 'stem cell' OR 'stem cell':ti,kw OR 'stem cells':ti,kw OR (stem NEAR/5 cell) OR (stem NEAR/5 cells) OR (progenitor NEAR/5 cell) OR (progenitor NEAR/5 cells) OR (embryo NEAR/5 cell) OR (embryos NEAR/5 cell) OR (embryonic NEAR/5 cell) OR (embryonal NEAR/5 cell) OR (embryo NEAR/5 cells) OR (embryos NEAR/5 cells) OR (embryonic NEAR/5 cells) OR (embryonal NEAR/5 cells) OR (fetal NEAR/5 cell) OR (foetal NEAR/5 cell) OR (fetal NEAR/5 cells) OR (foetal NEAR/5 cells) OR (umbilical NEAR/5 cell) OR ('bone marrow' NEAR/5 cells) OR ('cord blood' NEAR/5 cell) OR ('cord blood' NEAR/5 cells) OR (('neural stem' OR 'mesenchymal stem' OR 'mesenchymal stromal' OR 'mononuclear' OR 'mononuclear precursor' OR 'schwann' OR 'olfactory ensheathing' OR 'olfactory glia' OR 'oligodendrocyte precursor') AND ('cell'/exp OR cell OR 'cells'/exp OR cells)))

AND

('cerebrovascular accident'/mj OR stroke:ti,kw OR 'stroke' OR 'strokes' OR 'cerebrovascular accident' OR 'cerebrovascular accidents')

AND

'x-ray computed tomography'/exp/mj OR 'x-ray computed tomography':ti,kw OR 'neuroimaging'/exp/mj OR 'neuroimaging':ti,kw OR 'mri'/exp/mj OR 'mri':ti,kw OR 'magnetic resonance imaging'/exp/mj OR 'magnetic resonance imaging':ti,kw OR 'ct scan'/exp/mj OR 'ct scan':ti,kw OR 'cat scan'/exp/mj OR 'cat scan':ti,kw OR 'computerized tomographic imaging':ti,kw OR 'xray tomography':ti,kw OR 'ultrasonography'/exp/mj OR 'ultrasonography':ti,kw OR 'ultrasound'/exp/mj OR 'ultrasound':ti,kw OR 'single photon emission computed tomography computed tomography'/exp/mj OR 'single photon emission computed tomography computed tomography':ti,kw OR 'positron emission tomography computed tomography'/exp/mj OR 'positron emission tomography computed tomography':ti,kw OR 'diffusion tensor imaging'/exp/mj OR 'diffusion tensor imaging':ti,kw OR 'diffusion magnetic resonance imaging'/exp/mj OR 'diffusion magnetic resonance imaging':ti,kw OR 'functional mri'/exp/mj OR 'functional mri':ti,kw OR 'arterial spin labeling'/exp/mj OR 'arterial spin labeling':ti,kw OR 'single delay asl':ti,kw OR 'multi delay asl':ti,kw OR 'perfusion weighted imaging':ti,kw OR 'positron emission tomography'/exp/mj OR 'positron emission tomography':ti,kw OR 'single photon emission computed tomography'/exp/mj OR 'single photon emission computed tomography':ti,kw OR 'pet scan'/exp/mj OR 'pet scan':ti,kw OR 'spect':ti,kw

Scopus: ( ( TITLE ( ( "Stem Cells" OR "stem cell" ) ) OR AUTHKEY ( ( "Stem Cells" OR "stem cell" ) ) ) OR ( TITLE ( ( "cell transplantation" OR "stem cells" OR "Stem cell" OR "stem cells" OR "progenitor cell" OR "progenitor cells" OR "embryo cell" OR "embryos cell" OR "embryonic cell" OR "embryonal cell" OR "embryo cells" OR "embryos cells" OR "embryonic cells" OR "embryonal cells" OR "fetal cell" OR "foetal cell" OR "Fetal cells " OR "foetal cells" OR "umbilical cell" OR "bone marrow cells" OR "cord blood cell" OR "cord blood cells" ) OR ( ( "neural stem" OR "mesenchymal stem" OR "mesenchymal stromal" OR "mononuclear" OR "mononuclear precursor" OR "Schwann" OR "olfactory ensheathing" OR "olfactory glia" OR "oligodendrocyte precursor" ) AND ( cell OR cells ) ) ) ) )

AND

( TITLE-ABS-KEY ( "Stroke" OR "strokes" OR "Cerebrovascular Accident" OR "Cerebrovascular Accidents" ) )

AND ( TITLE-ABS-KEY ( "Neuroimaging" OR "Magnetic Resonance Imaging" OR "X-Ray Computed Tomography" OR "Ultrasonography" OR neuroimaging OR mri OR "magnetic resonance imaging" OR "CT scan" OR "CAT scan" OR "computerized tomographic imaging" OR "Xray tomography" OR ultrasonography OR ultrasound OR "Single Photon Emission Computed Tomography Computed Tomography" OR "Tomography, Emission-Computed, Single-Photon" OR "Positron Emission Tomography Computed Tomography" OR "Diffusion tensor imaging" OR "Diffusion Magnetic Resonance Imaging" OR "functional MRI" OR "arterial spin labeling" OR "single delay ASL" OR "multi delay ASL" OR "perfusion weighted imaging" OR "positron emission tomography" OR "single photon emission computed tomography" OR "PET scan" OR spect ) )
